# Editorial: Insights in public health policy: 2022

**DOI:** 10.3389/fpubh.2023.1227503

**Published:** 2023-08-01

**Authors:** Stefania Salmaso

**Affiliations:** Independent Researcher, Rome, Italy

**Keywords:** Tik Tok, personalized medicine, racism, hospital role, mathematical models, scientific publication, public health curricula

This Research Topics initiative aims to collect contributions on new insights, novel approaches, current challenges, and future perspectives in the field of Public Health Policy. Under this wide umbrella the RT includes manuscripts of different editorial types and topics, spanning from new communication tools in public health and personalized medicine to the *post-hoc* assessment of the performance of mathematical models for COVID-19 and the current challenges of scientific publishing.

Communication has been one of the most challenging issues during the COVID-19 pandemic. This original research article evaluates the use of Tik Tok in Shanghai as a visual tool for the rapid dissemination of public health messages, including to illiterate persons. In Shanghai, TikTok became the primary public information source during the public health emergency posed by the pandemic, with content mostly focused to uncertainty reduction, reassurance, and effectiveness of countermeasures. The study used the crisis and emergency risk communication (CERC) theory as a basis for the evaluation. While it is acknowledged that flexibility in communication strategies is very important, the study underlines the need for methods to assess and compare the performance and effects of different tools in various contexts.

Personalized medicine (PM) is currently recognized as a major new frontier in patient care, but its introduction in health settings means translating biotechnological advances to clinical routine and identifying key factors for successful implementation at the national level. A systematic review of this issue designs a framework to be used as a guide for developing future national PM implementation strategies and to rank countries and their current implementation strategies. The full implementation of PM poses significant ethical, legal, regulatory, organizational, and knowledge challenges. The proposed tool includes 11 domains comprising 37 individually quantifiable elements. Among the major current obstacle is the lack of interoperability of current data bases on genomic and molecular data, which have been designed so far only for research purposes. Even when these data are potentially available, they are not always included in the clinical decision-making process in the routine health care of patients.

Inequalities in health and health care are a highly relevant topic, exacerbated by the economic crisis, migration, and other current challenges in our society. A contribution in our RT argues for the need to establish a research agenda to explore the relationship between racism and health protection in order to build evidence that can be used to fight and dismantle racial inequities.

Hospitals are primarily devoted to diagnosis and treatment of individual patients with acute conditions. A manuscript in this RT raises the issue of the role that hospitals actually play (or can potentially play) in the health status of the community. The characteristics and limitations of such involvement are not well-defined and the many ways in which a hospital contributes to the health of its community and the impact on community health as well as the resources required are not easy to measure. The authors call for clarity on “how much” a hospital should do for its community, either as a moral or regulatory obligation or to achieve a change in the community's health status. They recommend that future governmental policies establish evidence-based, effective, measurable ways to involve hospitals in improving the health of communities.

A further opinion article discusses the gap between policy planners and analysts working at population level and managers and executives who work at local institutions, who administer resources to implement health policy and underlines the need for better coordination. The Authors compare the content of relevant educational programs in 11 leading universities in USA and observe that curricula diverged significantly, with students in health administration taking more traditionally business-oriented courses and students in public health, who are more likely to be involved in policy development, studying more public health and health policy-related subjects. Better initial interprofessional understanding and planning would result in more effective health systems that required less effort to implement and change. More research on the interdependence of policy makers and administrators is recommended to enhance the complementary roles of health care policy planners and health care managers.

During the recent pandemic most public health interventions were guided by scenarios designed with mathematical models. Initially available results were felt to overestimate the number of cases and deaths, often resulting in postponement of countermeasures. In USA, the initial estimate of 100,000 deaths was initially considered alarmistic, but the actual number of deaths attributable to COVID-19 is now over one million. As a fundamental tool to set the suitable response, it is important to continue to develop mathematical models and to assess their performance by comparing their results with the actual observations later on. The manuscript by Bowie and Friston assesses the validity of a 12-month projection estimating cases that would occur over the next year and discusses the factors that influenced the findings. The results of the dynamic model were found to underestimate the actual burden of the pandemic. The increase in transmissibility and the public's response, provide plausible explanations for why the model underestimated the 12-month predictions through October 2022. Similarly, the 2023 projection could also underestimate the predicted next wave of COVID-19 infection, and public health preparedness may well need to be reinforced.

An opinion paper by Vineis underlines the challenges of publishing scientific papers of good quality in a context where controversial and polarizing contents are fueled by non-scientific factors including commercial incentives. The pandemic “infodemic” has spilled over into the scientific literature as the pandemic rapidly evolved, and a great number of scientific papers have circulated without any adequate review. Additionally, the Author observes that the growing knowledge that health issues are closely linked to social, personal and behavioral characteristics has widened the public health area, requiring wider competences for the editors and reviewers. At the same time, the increasing awareness of the many issues faced by emerging economies and low-income countries requires that editors and reviewers have a greater understanding of the context of these settings and this represents a further challenge to the selection of good quality scientific papers. The Author concludes that we need to reaffirm the key values of scientific publishing, keeping in mind that the underlying values of quality and relevance are of greater importance than quantity and speed of publication.

## Author contributions

The author confirms being the sole contributor of this work and has approved it for publication.

